# Determining the Sterilization Doses under Hypoxia for the Novel Black Pupae Genetic Sexing Strain of *Anastrepha fraterculus* (Diptera, Tephritidae)

**DOI:** 10.3390/insects12040308

**Published:** 2021-03-30

**Authors:** Paloma Della Giustina, Thiago Mastrangelo, Sohel Ahmad, Gabriel Mascarin, Carlos Caceres

**Affiliations:** 1 Center for Nuclear Energy in Agriculture (CENA/USP), Piracicaba, São Paulo 13416-000, Brazil; pgdgiustina@usp.br (P.D.G.); piaui@cena.usp.br (T.M.); 2 Insect Pest Control Laboratory, Joint FAO/IAEA Centre of Nuclear Techniques in Food and Agriculture, A-2444 Seibersdorf, Austria; sohel.ahmad@iaea.org; 3 EMBRAPA Environment, Jaguariúna, São Paulo 13918-110, Brazil; gabriel.mascarin@embrapa.br

**Keywords:** sterile insect technique, irradiation, oxygen, lineage, south american fruit fly

## Abstract

**Simple Summary:**

The sterile insect technique is an environmentally-friendly method that can be used to manage populations of the South American fruit fly *Anastrepha fraterculus*, but its sterilizing doses have been determined only under normoxia. To maintain sterile male fly quality without jeopardizing sterility, a strategy of applying the radiation dose under an oxygen-reduced atmosphere is commonly used. Therefore, this study tested a range of gamma radiation doses under hypoxia on pupae from a bisexual strain and a novel genetic sexing strain (GSS) of *A. fraterculus*. Four types of crosses between irradiated flies under hypoxia and nonirradiated flies of the bisexual strain were set to assess sterility for each strain and radiation dose. For males from both strains, the effect of radiation dose on the percentage of egg hatch, egg-to-pupa recovery, and recovery of adults were determined. A dose of 74 Gy reduced egg hatch by 99% regardless of the male strain and was considered the sterilizing dose. The fertility of irradiated females and development of their offspring were severely affected, even at low doses under hypoxia, and no eggs were laid with doses above 50 Gy.

**Abstract:**

A common strategy used to maintain sterile fly quality without sacrificing sterility is to irradiate the insects under an oxygen-reduced atmosphere. So far, sterilizing doses for the South American fruit fly *Anastrepha fraterculus* have only been determined under normoxia. Our study reports for the first time the dose-sterility response under hypoxia for two different *A. fraterculus* strains. The pupae were derived from a bisexual strain (a Brazilian-1 population) and a recently developed genetic sexing strain (GSS-89). Two hours prior to irradiation, pupae were transferred to sealed glass bottles and irradiated when oxygen concentration was below 3%. Four types of crosses with nonirradiated flies of the bisexual strain were set to assess sterility for each radiation dose. For males from both strains, Weibull dose–response curves between radiation doses and the proportion of egg hatch, egg-to-pupa recovery, and recovery of adults were determined. The GSS males revealed high sterility/mortality levels compared to males from the bisexual strain at doses < 40 Gy, but a dose of 74 Gy reduced egg hatch by 99% regardless of the male strain and was considered the sterilizing dose. The fertility of irradiated females was severely affected even at low doses under hypoxia.

## 1. Introduction

Of the 250 species of the genus Anastrepha Schiner present in the Neotropics, the South American fruit fly *Anastrepha fraterculus* (Wiedemann) is one of the most economically important tephritids [1]. Actually, the nominal *A. fraterculus* is a complex of cryptic species that comprises so far eight recognized morphotypes throughout Central and South America, which may assume pest status depending on the geographical location [2]. The Brazilian-1 morphotype prevails in Argentina and southern Brazil and is considered a severe pest of economic importance for more than 40 types of fruits [3]. For example, the economic loss during the Brazilian 2019/2020 citrus harvest due to fruit drop caused by fruit fly species (ca. 820.1 million tons) was estimated at USD $92 million [4]. Therefore, the availability of modern environment-friendly control methods, such as the Sterile Insect Technique (SIT), is very important for fruit growers in these countries.

For the past three decades, many efforts have been made to meet the prerequisites required to implement the SIT against *A. fraterculus* [5]. Colonies of this fly have been successfully maintained under semi mass-rearing conditions in Argentina and Brazil [6,7]. A protocol to sterilize the mass-reared flies is another essential technical requirement in an SIT program. Traditionally, pupae from tephritids have been sterilized by ionizing radiation emitted by ^60^*Co* or particle accelerators [8]. Gamma radiation, e-beam, or X-rays, can induce double strand breaks in DNA, leading to the production of dominant-lethal mutations (DLMs) in reproductive cells and rendering the adult flies sterile [9]. Although sterility increases with increasing doses, so does the negative effects of the radiation on the quality of the insect in terms of adult emergence, flight, pheromone production, longevity, and mating competitiveness [10,11,12]. Negative side effects from complete sterility doses on quality control parameters, such as lower longevity and sexual performance, have been reported for some mass-reared *Anastrepha* species [13,14,15,16,17,18,19].

To maintain better sterile male fly quality without jeopardizing sterility, irradiating the insects in an oxygen-reduced atmosphere (hypoxia) has been a common strategy in fruit fly facilities for more than 49 years [10]. This follows the discovery of the radiological protective effects of nitrogen on the competitiveness of sterile Mediterranean fruit fly *Ceratitis capitata* (Wiedemann) [20]. Tephritidae pupae, one or two days prior to adult emergence, are usually sealed in 2–4 L plastic bags and held at low temperatures (<20 °C) for at least 1 h before irradiation, and during this period the pupae exhaust most of the oxygen from the bags [21]. The resulting hypoxia reduces the oxidative damages to genomic and other organic macromolecules during irradiation, as in the absence of oxygen the free radicals produced in body tissues from the split of cellular water and gaseous oxygen by ionizing radiation can be better neutralized by antioxidants and hydrogen radicals [8]. Besides reducing unwanted irradiation damages, hypoxia may also delay adult emergence and, thus, give more time for the sealed bags to be shipped to emergence facilities or release areas [22,23].

Dose response studies have been carried out for *A. fraterculus* only under normoxia conditions, i.e., a normal oxygen level of 21%. Full sterility can be obtained in both males and females with a dose of 70 Gy [16]. A lower dose of 40 Gy of gamma or X-rays administered to pupae 72 h before emergence renders the male sterile by 99% and results in atrophy of female ovaries [24]. As the sensitivity of the pupae to radiation exposure is reduced under hypoxia conditions, higher doses are usually required to generate comparable sterility levels [8,25]. Therefore, further radiosterilization studies are necessary to evaluate the effects of hypoxia or anoxia condition prior to and during irradiation of *A. fraterculus* pupae. 

As the simultaneously release of sterile males and females may reduce the efficiency of the SIT due to the occurrence of matings between sterile pairs [24,26,27], most fruit fly SIT programs seek to invest in the development of genetic sexing strains (GSS) that allow the release of only males. This, however, requires a mechanism to sort the sexes at some stage of their development [9,10,28]. Recently, the Insect Pest Control Laboratory (IPCL), Joint FAO/IAEA Centre of Nuclear Techniques in Food and Agriculture, Seibersdorf, Austria, developed a genetic sexing strain (GSS) of *A. fraterculus* (named GSS-89) derived from a Brazilian-1 morphotype population that was based on a pupa color dimorphism, i.e., males emerge from brown pupae while the females emerge from black pupae [29]. Due to the presence of a chromosomal translocation between the “Y” chromosome and the autosome that carry the wild type allele of the gene responsible for the pupae color, the GSS-89 males show a lower fertility as compared with the Vacaria strain, a bisexual strain of the Brazilian-1 morphotype that is reared for SIT-projects in Brazil (e.g., 54% and 88% fertility, respectively) [24,29]. The dose responses of the novel GSS are still unknown.

This study aimed to determine the sterilization doses for the GSS-89 in comparison with the wild type bisexual Vacaria strain when their pupae are irradiated under hypoxia conditions, as well to assess the effects of hypoxia on the development and biological parameters from the offspring of both strains.

## 2. Materials and Methods

### 2.1. Anastrepha fraterculus Colonies

The *A. fraterculus* pupae used for the sterilization tests under hypoxia were derived from colonies of a bisexual strain and the GSS-89 maintained at the IPCL. The bisexual strain belonged to the Brazilian-1 morphotype originally collected from infested guavas in the locality of Vacaria, Brazil (28°31′08′′ S, 50°52′18′′ W) in 2015 and since then maintained at the IPCL for 50 generations. The GSS-89 was developed from the same laboratory strain of the Vacaria population in 2016 and the sexing system was developed by the radio-induction of a reciprocal translocation between the Y chromosome and the autosome that carries the wild type allele of the black pupae (bp) gene [29]. The GSS-89 colony was reared for 36 generations. The adults were kept in cages and provided ad libitum with water and a diet containing a mixture at a 3:1 ratio of sugar and hydrolysate enzymatic yeast (MP Biomedicals^TM^). Larvae from both strains were reared on a standard carrot diet [30]. The colonies were maintained in rooms with controlled environmental conditions (24 ± 1 °C, 60% RH, and 14:10 h light:dark photoperiod).

### 2.2. Irradiation Procedures

All pupae were irradiated 24 h or less before adult emergence in a ^60^*Co* irradiator (GammaCell-220, MDS Nordion, Ottawa, ON, Canada) that had an activity of 203.8 TBq and a dose rate of 80 Gy/min. Two hours prior to the irradiation, pupae were transferred to sealed glass bottles (25 mL) and the O_2_ and CO_2_ levels were monitored at 22 °C using a gas-sensor device (Dansensor^®^ CheckMate3, Ringsted, Denmark). When the oxygen concentration was lower than 3% inside the vials, they were taken to the irradiator. To provide dose uniformity, the sealed vials were placed at the center of the radiation chamber. For each exposure, dosimetry was performed using the Gafchromic^®^ dosimetry system [31]. Pupae were treated with eight doses (30, 40, 50, 60, 70, 80, 90, and 100 Gy) in addition to the untreated control. After irradiation, the vials were opened and treated pupae were placed in cages until adult emergence. Pupal cohorts from different generations were used for the exposures.

### 2.3. Evaluation of Sterilization under Hypoxia

Emerged flies of the GSS-89 and the bisexual strain, henceforth named GSS and VAC, respectively, were manually sexed and the sterility was assessed for each radiation dose on four types of crosses: (1) 50 irradiated GSS males × 50 non-irradiated VAC females; (2) 50 irradiated VAC males × 50 non-irradiated VAC females; (3) 50 non-irradiated VAC males × 50 irradiated GSS females; and (4) 50 non-irradiated VAC males × 50 irradiated VAC females. Two cages that contained 50 pairs of non-irradiated flies from both strains were kept separately as untreated controls. The flies were kept in 7 L cylindrical acrylic cages and fed *ad libitum* with the abovementioned mixture of sugar and hydrolyzed yeast (3:1), while water was provided in plastic containers with a strip of cellulose sponge cloth. Females oviposited their eggs through the netting on the top of the cage into a water-filled Petri dish with a black silicon panel. The oviposition device was deployed every morning, and after 3 h, the eggs were collected and the oviposition devices cleaned. Each cross was replicated three times and eggs were collected five times from each cage.

From each cage, 200 eggs were counted and lined on a black fabric strip placed over a moistened sponge in a Petri dish and transferred to an identified Petri dish filled with carrot diet [30]. Egg hatch was assessed after 7 days (25 ± 1 °C and 70% RH), and the fabric strip removed. After larval development, the pre-pupae crawled out of the Petri dishes and were collected in a container with sawdust to pupate. The sawdust was sifted after 7 days and the pupae from each treatment were counted and placed in Petri dishes. The percentages of egg-to-pupa recovery ((n° pupae ÷ 200) × 100) and recovery of adults ((n° emerged adults ÷ 200) × 100) were assessed based on the number of pupae and adults obtained from each Petri dish. The parameter of adult emergence was obtained according to the FAO/IAEA/USDA Manual [32], while sex ratio was calculated as the division of the number of females by the total number of males and females [24]. A total of 15 replicates were performed for each treatment.

### 2.4. Data Analysis

For data modeling, the relationships between the proportion of egg hatch, egg-to-pupa recovery and recovery of adults for males were analyzed with functions *drm()* and *mselect()* of the “DRC” package in R (Dose–Response Curves, Version 3.0-1) [33] in order to find the best fitted models by comparing the log-likelihood values, Akaike Information Criteria (AIC), lack of fit and residual variance of all models. The datasets were fitted to the non-linear 4-parameter Weibull model (W1.4), $y=\;c+\left(d-c\right)\exp\left(-\exp\left(b\left(\log\left(x\right)-\log\left(e\right)\right)\right)\right)$, with binomial distribution for errors and including as fixed effects the radiation dose and fly strain in the linear predictor. For this model, *d* is the upper limit, *c* is the lower limit (which here it was not significant and tended to zero at the highest doses of 90–100 Gy), *b* is the slope, *e* is the median effective irradiation dose (ED_50_) and *x* is the radiation dose (Gy). Doses providing sterility levels of 50, 90, and 99% were determined with their respective 95% confidence intervals and then the estimated doses from both strains were compared using Student’s *t*-test (*p* < 0.05). All graphs were made using the ggplot2 package [34] in the statistical environment R [35].

For each strain, the results related to emergence of adults (%) and sex ratio (♀/♂ + ♀) were submitted to non-parametric Kruskal–Wallis analysis of variance, and pairwise comparisons of means were performed by the Mann–Whitney *U* test when necessary. As eggs were obtained only from females irradiated at doses lower than 50 Gy, there was not sufficient data points for adequately fitting dose–response models, and the Kruskal–Wallis analysis of variance was applied to the biological data from crosses with irradiated females, and the Mann–Whitney *U* test to compare means. All analyses were performed in R [35].

## 3. Results

### 3.1. Irradiation of Males under Hypoxia

**Figure 1:**
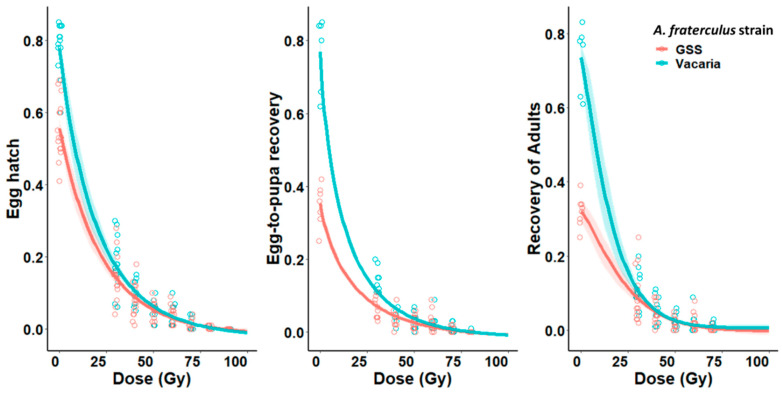
Weibull dose–response curves with 95% confidence intervals for three biological parameters resulting from crosses between fertile female *Anastrepha fraterculus* and males from a genetic sexing strain (GSS) and a bisexual strain (Vacaria) irradiated under hypoxia.

Table 1, Table 2 and Table 3 present the means (± standard errors) from the biological parameters obtained from crosses between fertile VAC females and males of the bisexual strain and GSS irradiated under hypoxia. The relationship between irradiation dose and the proportion of egg hatch, egg-to-pupa recovery and recovery of adults were determined (Table 1 and Figure 1). The mean egg hatch and pupal and adult recovery decreased significantly with increasing radiation doses. No egg hatch was observed with doses of 90 and 100 Gy (Table 1). The four-parameter Weibull model was the best fitting model for describing the dose response curves for egg hatch, egg-to-pupa recovery and adult recovery (Figure 1). The lower fertility of untreated GSS males (i.e., egg hatch around 50% and recovery of pupae and adults below 40%) and a possible combination between this natural sterility and irradiation effects resulted in a distance between the curves of the two strains when the males were treated with doses lower than 40 Gy. However, the trend between the two strains was similar and the curves tended to overlap at higher doses (Figure 1).

Estimated doses based on the Weibull model giving 50 (D_50_), 90 (D_90_), and 99% (D_99_) sterility or mortality of pupae and adults, with lower and upper confidence limits (95%), are shown in Table 2. Considering the three biological parameters, the mean estimated D_50_ varied between 2.2 and 8.1 Gy, but it was not possible to assess egg-to-pupa recovery and recovery of adults for the GSS as the initial values for those parameters were lower than 40% (e.g., 35.5% and 32.1%, respectively) (Table 1). The estimated D_90_ ranged from 23.1 to 41.1 Gy among parameters, while the D_99_ varied from 62.9 to 74.1 Gy (Table 2). Some of the 95% confidence intervals overlapped, resulting in no difference for some estimated doses of the two strains. For example, D_99_ for egg hatch was ca. 74 Gy for both strains. Significant differences (*p* < 0.05) between the two strains for each estimated dose were found only in D_50_ for egg-hatch (*t* = 8.5; *p* < 10^−3^), D_90_ (*t* = 57.8; *p* < 10^−3^) and D_99_ (*t* = 3.7; *p* = 0.0002) for egg-to-pupa recovery, and in D_90_ for recovery of adults (*t* = 2.1; *p* = 0.04).

Emergence of adults and sex ratio were also significantly affected (*p* < 0.05) by radiation dose applied under hypoxia. For the two strains, the percentage of adult emergence was lower than 70% with doses above 50 Gy, and treating the males with a dose of 80 Gy resulted in significant differences between untreated control males and VAC (*Z* = 3.6; *p* < 10^−3^) and the GSS (*Z* = 3.03; *p* = 0.002) males. For VAC, the mean values of sex ratio were around 0.4 until 60 Gy, with a significant difference between the control and the mean at 80 Gy (*Z* = 3.5; *p* < 10^−3^). The sex ratio values varied more for the GSS, with the mean ranging from 0.02 to 0.50 and a significant difference between the control and the treatment at 70 Gy (*Z* = 4.4; *p* < 10^−3^).

### 3.2. Irradiation under Hypoxia of Females

The radiation treatments under hypoxia affected significantly the fertility and development of offspring even at low doses (Table 4). Females from both strains irradiated with 50 Gy were not able to lay eggs, and females treated with a dose of 40 Gy showed a mean egg-to-pupa recovery and recovery of adults of 0.1–0.2%. GSS females from the control group presented a fertility similar to VAC females (~74–77%) when crossed with fertile VAC males. However, viability of eggs produced by the females of the two strains irradiated with 40 Gy was at least three times lower as compared with the untreated control (VAC: *Z* = 4.7, *p* < 10^−3^; GSS: *Z* = 3.04; *p* = 0.002). Eggs were obtained from three treatments (i.e., control, 30 and 40 Gy) and therefore, it was not possible to fit dose–response curves to the biological parameters of females, although Kruskal–Wallis tests indicated that all treatments differed significantly (*p* < 10^−3^) (Table 4).

The mean values of adult emergence and sex ratio for both strains remained high up to the dose of 30 Gy (>80% and >0.3, respectively) (Table 4). The differences between the control and the dose with 40 Gy were significantly for both percent of adult emergence (VAC: *Z* = 3.6, *p* < 10^−3^; GSS: *Z* = 3.8; *p* < 10^−3^) and sex ratio (VAC: *Z* = 3.5, *p* < 10^−3^; GSS: *Z* = 3.8; *p* < 10^−3^).

## 4. Discussion

The assessment of the effects of hypoxia during a radiation treatment is very important for the potential implementation of the SIT against *A. fraterculus* as low oxygen levels may reduce negative effects of irradiation [10]. The mechanisms related to the hypothesis of oxidative stress are still being elucidated and involve biochemical, antioxidant and cellular responses [36]. Hypoxia conditions in tissues can trigger an increase in total antioxidant response, and a resulting decrease in oxidative damage to macromolecules [37,38]. Furthermore, Lopez-Martinez and Hahn [39] presented indications of an increased antioxidant enzyme activity in *Anastrepha suspensa* (Loew) for at least 24 h after anoxic conditioning of adults just prior to emergence, which was associated with a reduction in post-irradiation oxidative damage up to 10 days after irradiation and a greater male sexual competitiveness. This was one study among several others that have demonstrated that an increase in antioxidant defense, even transiently during the irradiation process, can produce long-lasting positive effects on sterile male insect performance [13,40].

Even though the benefits of low-oxygen conditioning on irradiation and insect performance for the SIT have been known since the 1970s [20,41,42], studies so far dedicated to the radio-sterilization of *A. fraterculus* have been carried out under normoxia conditions [16,24]. Therefore, a range of gamma radiation doses under hypoxic conditions were tested in this study on *A. fraterculus* pupae from two different strains to assess any possible radiological protection effect on male and female adults and on the development of their offspring.

Irradiation decreased both fertility of adults and survival of the immature stages for the different strains. As the radiation-induced DLMs act by mainly causing early zygotic death, egg hatch is the most commonly used parameter to estimate sterilizing doses in tephritids [8]. Therefore, sterilizing effects can be detected by a significant reduction in numbers of descendants after a crossing with a sterile male. The values of egg-to-pupa recovery reported by Meza et al. [29] for the VAC strain and the GSS-89 (e.g., 73 ± 1.3% and 29.8 ± 4.6%, respectively) were similar to those observed in our control groups (Table 1). However, as doses increase, fewer individuals survived to late developmental stages (Figure 1). Less than 1% of adult flies were recovered from eggs laid by fertile VAC females crossed with VAC males irradiated with 80 Gy or GSS males that received 70 Gy (Table 1). These results indicate that random DLMs can lead to the death of the offspring at later stages of development. The same effect has also been observed for other insects [9,43]. Orozco-Davila et al. [44] have likewise reported low recovery of flying adults from eggs laid by wild *Anastrepha ludens* (Loew) females crossed with the GSS Tapachula-7 males irradiated with relative low doses of gamma radiation.

Based on the Weibull dose–response curves for egg hatch data (Table 1 and Table 2), the estimated dose of 74 Gy administered to male pupae from both strains under hypoxia induced 99% sterility in untreated females. That dose is almost twice the recommended radiation dose (ca. 40 Gy) to induce 99% sterility in the same bisexual strain of *A. fraterculus* under normoxia [24]. This phenomenon of higher doses being required under hypoxia to induce comparable reproductive sterility than in normoxia conditions is expected, since the radiation-induced reproductive damage is reduced during irradiation under a low-oxygen environment [8].

High sterilizing doses under hypoxia have been reported for Lepidoptera [45], tsetse flies [46], drosophilids [47], and tephritids [8,48]. For *C. capitata*, the sterilizing doses under normoxia are between 70 and 100 Gy, while under hypoxia they have been estimated at between 90 and 160 Gy [49,50,51,52,53]. In the genus *Anastrepha*, pupae from *A. ludens* and *Anastrepha obliqua* (Macquart) can be sterilized under hypoxia with 70–80 Gy, but under normoxia lower doses are required for the same species (i.e., 30–40 Gy and 40–60 Gy, respectively) [44,54,55,56,57,58]. For *A. suspensa*, the sterilizing doses under normal and low-oxygen conditions are 50 and 70 Gy, respectively [13,15]. In *Anastrepha serpentina* (Wiedemann), the doses of 40 and 80 Gy induce 99.9% sterility in adult flies when irradiated as old pupae under normoxic or hypoxic-conditioning periods, respectively [59,60].

Our results suggest the existence of small differences between the sterilization of the males from both strains at low doses (Figure 1 and Table 2). While the fertility of the bisexual strain was around 80%, the natural fertility of the GSS males was 51.5% (which is equivalent to 48.5% natural sterility) (Table 1). These results suggest that such degree of inherent sterility may be a result of the genetic translocation present in the GSS males, which has also been observed in other genetic sexing strains of fruit flies, such as the VIENNA-8 *tsl* strain of *C. capitata* and Tapachula-7 of *A. ludens* [9,43,44].

In females from the two strains, fertility was severely reduced with increasing radiation doses (Table 4). Even under hypoxia, females required much lower doses than males to achieve the same sterility, confirming the female’s higher radiation sensitivity. A dose of 50 Gy under hypoxia was required to obtain complete female sterility, whereas under normoxia a dose of 25 Gy was sufficient to cause complete ovarian atrophy in VAC females [24]. The eggs laid by the irradiated females showed some evidence of DLM as the mean egg-to-pupa recovery was lower than 20%. With the current available sexing system for *A. fraterculus* [29], the determination of optimal sterilizing doses for females has lost practical relevance, as only males of the GSS can be released in future SIT applications in the field. 

However, in a fruit fly free-area, 100% sterility may be required for any released female of a bisexual strain [8]. Rull et al. [17] proposed a strategy to release males irradiated with doses that induce 99% sterility (e.g., 74 Gy in our case) during the early suppression phase, when more competitive males would provide greater induction of sterility into relatively large wild populations, and release completely sterile flies (irradiated with 80–90 Gy) in pest free-areas. In any of the SIT application scenarios, the *A. fraterculus* females irradiated with 70–90 Gy would be fully sterile regardless of the strain.

Despite the protective effects of irradiation under hypoxia, special attention must be given to two parameters: the time under hypoxia before irradiation and temperature during hypoxia. Long exposures to oxygen deprivation at high temperatures can be deleterious for the quality of flies [17,61,62]. In view of these results, packing guidelines of tephritids have recommended a temperature of 15–20 °C during irradiation under hypoxia and shipping [21]. Further research with the interaction between temperature and hypoxic tolerance of different strains would be necessary before the adoption of hypoxic procedures in the management of *A. fraterculus* pupae for a SIT program.

Our findings have demonstrated that, like in other fruit fly species, irradiation under hypoxia can be adopted as a protocol for the sterilization of an *A. fraterculus* GSS. However, additional research should be conducted to complement the understanding of the effects of low-oxygen atmosphere on the biology of different *A. fraterculus* strains. The sterilizing doses under hypoxia for females of bisexual strains could be determined using a wider range of low radiation doses. Mating studies under semi-field conditions must also be performed to assess the competitiveness of males irradiated under hypoxia against males sterilized under normoxia and wild males when trying to mate with wild females. Even so, our study brings significant contributions related to sterilizing doses, which can be used for the development of a future protocol to irradiate pupae of different *A. fraterculus* strains under hypoxic conditions.

## 5. Conclusions

Our study reports for the first time the dose-sterility response under hypoxia for a new black pupae GSS and a bisexual strain of *A. fraterculus*. The dose of 74 Gy applied under hypoxic conditions can induce 99% sterility in males from both strains, while complete sterility can be achieved with 80–90 Gy.

## Figures and Tables

**Table 1:** Biological parameters (mean ± SE) from crosses between fertile females and irradiated *Anastrepha fraterculus* males from a bisexual strain (VAC) and a genetic sexing strain (GSS) under hypoxia.

Dose (Gy)	Biological Parameters					
	Egg Hatch (%)		Egg-to-Pupa Recovery (%)		Recovery of Adults (%)	
	VAC	GSS	VAC	GSS	VAC	GSS
**0**	80.3 ± 2.8	51.5 ± 1.2	76.6 ± 4.1	35.5 ± 1.9	73.5 ± 3.8	32.1 ± 1.5
**30**	18.03 ± 2.1	13.9 ± 1.6	12.1 ± 1.5	9.7 ± 1.8	10.9 ± 1.3	9.2 ± 1.7
**40**	9.3 ± 0.8	7.9 ± 1.2	5.2 ± 0.9	4.4 ± 0.8	4.8 ± 0.8	4.0 ± 0.7
**50**	5.2 ± 0.7	5.2 ± 0.7	2.6 ± 0.5	2.4 ± 0.5	2.3 ± 0.4	2.0 ± 0.4
**60**	3.9 ± 0.7	3.9 ± 0.6	2.03 ± 0.5	2.4 ± 0.7	1.7 ± 0.6	1.9 ± 0.6
**70**	1.7 ± 0.4	1.1 ± 0.3	0.73 ± 0.3	0.4 ± 0.2	0.6 ± 0.2	0.2 ± 0.15
**80**	0.13 ± 0.1	0.17 ± 0.06	0.07 ± 0.05	0	0.03 ± 0.03	0
**90**	0	0	0	0	0	0
**100**	0	0	0	0	0	0
**Weibull model **	y = (0.78)exp(exp(0.92(log(x) − log(20.6))))	y = (0.55)exp(exp(0.96(log(x) − log(23.1))))	y = (0.77)exp(exp(0.77(log(x) − log(13.9))))	y = (0.35)exp(exp(0.82(log(x) − log(19.1))))	y = (0.74)exp(exp(1.18(log(x) − log(17.1))))	y = (0.32) exp(exp (1.26 (log(x) − log(24.7))))

**Table 2:** Radiation doses (Gy) and their 95% confidence intervals (CIs) estimated from the 4-parameter Weibull model for selected sterility levels of *Anastrepha fraterculus* males from a bisexual strain (VAC) and a genetic sexing strain (GSS) irradiated under hypoxia.

Parameter/Strain		D_50_ (95% CI) ^†^		D_90_ (95% CI)		D_99_ (95% CI)	
**Egg hatch**	**VAC**	8.1 (5.7; 10.6)	t = 8.45 *p* < 10^−3^	41.1 (37.02; 45.1)	t = 1.4 ^ns §^ *p* = 0.15	74.05 (57.9; 90.2)	t = 0.0043 ^ns^ *p* = 0.99
	**GSS**	2.2 (0.9; 3.4)		37.4 (33.9; 40.8)		74.1 (58.9; 89.3)	
**Egg-to-Pupa Recovery**	**VAC**	4.5 (4.3; 4.8)	---	32.3 (32.2; 32.5)	t = 57.8 *p* < 10^−3^	68.5 (66.8; 70.2)	t = 3.7 *p* = 0.0002
	**GSS**	NE ^‡^		23.1 (22.8; 23.4)		64.6 (63.2; 65.9)	
**Recovery of Adults**	**VAC**	7.7 (4.3; 11.1)	---	31.3 (29.3; 33.3)	t = 2.1 *p* = 0.04	68.6 (51.1; 86.1)	t = 0.46 ^ns^ *p* = 0.64
	**GSS**	NE		27.6 (24.5; 30.7)		62.9 (45.4; 80.5)	

^†^ D = dose (Gy) that induces 50, 90, or 99% sterility and its 95% confidence interval. ^‡^ NE: Not estimated. ^§^ ns = comparison between estimated doses of the two strains not significant by the Student’s *t*-test (*p* > 0.05).

**Table 3:** Means (± SE) of adult emergence and sex ratio of flies originated from crosses between fertile females and *Anastrepha fraterculus* males from a bisexual strain (VAC) and a genetic sexing strain (GSS) irradiated under hypoxia. Total numbers of scored adults are in parentheses.

Dose (Gy)	Biological Parameters			
	Adult Emergence (%)		Sex Ratio (♀/♂ + ♀)	
	VAC	GSS	VAC	GSS
**0**	96.1 ± 1.1	90.8 ± 1.5	0.4 ± 0.01 (*n* = 1323)	0.44 ± 0.02 (*n* = 770)
**30**	92.04 ± 2.1	92.6 ± 2.4	0.4 ± 0.03 (*n* = 329)	0.46 ± 0.04 (*n* = 275)
**40**	93.5 ± 1.5	83.9 ± 7.0	0.4 ± 0.1 (*n* = 143)	0.5 ± 0.05 (*n* = 120)
**50**	83.9 ± 6.6	69.9 ± 9.7	0.39 ± 0.1 (*n* = 68)	0.28 ± 0.07 (*n* = 60)
**60**	61.8 ± 9.7	66.1 ± 9.5	0.38 ± 0.1 (*n* = 50)	0.38 ± 0.1 (*n* = 58)
**70**	40.6 ± 11.9	17.8 ± 9.7	0.19 ± 0.1 (*n* = 18)	0.02 ± 0.01 (*n* = 7)
**80**	6.7 ± 6.6	0	0.07 ± 0.06 (*n* = 1)	NE
**90**	0	0	NE ^†^	NE
**100**	0	0	NE	NE
**Kruskal-Wallis Analysis of Variance**	χ^2^ = 77.4 *p* < 10^−3^	χ^2^ = 83.6 *p* < 10^−3^	χ^2^ = 57.9 *p* < 10^−3^	χ^2^ = 86.5 *p* < 10^−3^

^†^ NE: not estimated because no females were obtained.

**Table 4:** Biological parameters (mean ± SE) from crosses between fertile male *Anastrepha fraterculus* from a bisexual strain (VAC) and females irradiated under hypoxia from the same strain and a genetic sexing strain (GSS). Total numbers of scored adults are in parentheses.

Strain	Dose (Gy)	Biological Parameters				
		Egg Hatch (%)	Egg-to-Pupa Recovery (%)	Recovery of Adults (%)	Adult Emergence (%)	Sex Ratio (♀/♂ + ♀)
**VAC**	**0**	78.3 ± 1.6	76.6 ± 2.6	73.5 ± 3.3	96.1 ± 0.7	0.4 ± 0.01 (*n* = 1324)
	**30**	27.2 ± 3.9	18.9 ± 2.4	18.0 ± 2.2	95.6 ± 1.5	0.5 ± 0.03 (*n* = 540)
	**40**	10.2 ± 5.1	0.1 ± 0.05	0.1 ± 0.05	6.7 ± 6.6	0.07 ± 0.06 (*n* = 3)
	**50**	0	0	0	0	NE ^†^
	**60**	0	0	0	0	NE
**Kruskal–Wallis Analysis of Variance**		χ^2^ = 63.6 *p* < 10^−3^	χ^2^ = 54.2 *p* < 10^−3^	χ^2^ = 59.5 *p* < 10^−3^	χ^2^ = 55.9 *p* < 10^−3^	χ^2^ = 55.8 *p* < 10^−3^
**GSS**	**0**	74.7 ± 1.9	73.1 ± 2.5	69.3 ± 2.8	94.7 ± 1.4	0.39 ± 0.01 (*n* = 1662)
	**30**	16.9 ± 2.7	14.3 ± 2.7	13.6 ± 2.6	80.1 ± 8.7	0.42 ± 0.06 (*n* = 407)
	**40**	26.1 ± 10.3	0.2 ± 0.11	0.2 ± 0.1	6.7 ± 6.6	0.02 ± 0.01 (*n* = 6)
	**50**	0	0	0	0	NE
	**60**	0	0	0	0	NE
**Kruskal–Wallis Analysis of Variance**		χ^2^ = 54.3 *p* < 10^−3^	χ^2^ = 55.3 *p* < 10^−3^	χ^2^ = 54.3 *p* < 10^−3^	χ^2^ = 50.5 *p* < 10^−3^	χ^2^ = 50.7 *p* < 10^−3^

^†^ NE: not estimated because no females were obtained.

## Data Availability

Data available within the article.
